# A pilot study for development of a pulmonary function test induction jacket to automate effort in performing the forced vital capacity manoeuvre

**DOI:** 10.1038/s41598-023-34930-1

**Published:** 2023-05-17

**Authors:** Prashant Rajdeep, Ketaki Poorey, R. K. Patel, E. R. Oommen

**Affiliations:** 1 Department of Physiology, Medical College Baroda, The Maharaja Sayajirao University of Baroda, Vadodara, Gujarat 390001 India; 2 Department of Physiology, National Institute of Medical Sciences and Research, NIMS University, Jaipur, Rajasthan 303121 India; 3 Department of Physiology, Parul Institute of Medical Sciences & Research Parul Sevashram Hospital, Parul University, Vadodara, Gujarat 391760 India

**Keywords:** Biological techniques, Biophysics, Physiology, Health care, Medical research

## Abstract

The pulmonary function test (PFT) induction jacket was invented to make the process of performing the forced vital capacity (FVC) manoeuvre with a computerized spirometer effortless and productive for both the patient and the medical practitioner. The jacket is composed of three layers of PVC material sealed together to form a single jacket with two chambers. The inner chamber is formed between the inner layer and the middle layer, in which cold water at a temperature of 10 °C is circulated using a connected water unit when triggered. Similarly, the outer chamber is formed between the middle layer and the outer layer, in which air is pressurized using a connected air unit. Thirty volunteers performed the FVC manoeuvre with and without wearing the jacket. There was no difference between the results in spirometry parameters in the participants without a jacket and those with a jacket. However, use of the jacket significantly reduced the number of trials the participants had to undergo to perform spirometry. The jacket automated the FVC manoeuvre by triggering a physiological inspiratory gasp using cold water and circumscribing pressurized air for expiration. Additionally, subsequent advancements in the jacket have been suggested.

## Introduction

Over the last few years, the prevalence of lung function abnormalities has led to several mass testing programs and various health surveillance programs around the world. Pulmonary function tests (PFTs) have been widely used for diagnostic and research purposes in pulmonary medicine, otolaryngology, sports medicine, preoperative investigation and physiology^1,2^. PFTs measure different lung volumes and capacities, which in turn helps diagnose and classify obstructive or restrictive lung pathology. PFTs aid in assessing lung function, which has become a crucial prognostic tool in the COVID-19 pandemic era, where patients are presenting with post-COVID lung function impairment of a variety of types as well as percentages^3^.

PFTs can be performed by two methods: spirometry (a device with a mouthpiece hooked up to a small electronic machine) and plethysmography (an airtight box that looks like a short, square telephone booth), of which spirometry is the most common, as the instrument is portable^4^.

The spirometer measures the maximal volume of air that an individual can inspire and expire with maximal effort, thus capturing the signal of either volume or flow as a function of time. Among the various parameters measured by spirometer, forced vital capacity (FVC) is the most relevant. FVC is the volume delivered during an expiration made as forcefully and completely as possible starting from full inspiration^5^.

Measuring FVC with a spirometer requires patients to follow a specific breathing protocol known as the FVC manoeuvre, which has four distinct phases: (1) maximal inspiration, (2) a “blast” of expiration, (3) continued complete expiration for a maximum of 15 s, and (4) inspiration at maximal flow back to maximum lung volume. Moreover, patients must perform well a number of times to obtain acceptable and reproducible results.

The American Thoracic Society (ATS) and the European Respiratory Society (ERS) jointly published the guidelines and technical standards for conducting spirometry. The guidelines are updated periodically to consider evolutions in best practices and improvements in instrumentation and computational capabilities, together with new research studies and enhanced quality assurance approaches. The most recent update was published in 2019. According to the update, the “operator” is the person conducting the test and has the most important responsibility to observe and engage with the patient to achieve optimal results, which requires a combination of training and experience. However, operator training and attainment and maintenance of competency requires short-term follow-up and supplementary training, which may not be available in all countries^6^. Additionally, Ruppel GL and Enright PL observed, “There are 3 key elements to obtain high quality pulmonary function data: accurate and precise instrumentation, a patient/subject capable of performing acceptable and repeatable measurements, and a motivated technologist to elicit maximum performance from the patient. In the realm of standardization, the technologist has received the least attention^7^”. The 2019 ATS/ERS technical statements have also summarized the patient experience and indirectly state that 20% of the respondents found the degree of difficulty in performing the PFT neither completely nor mostly acceptable. Additionally, 31% said that the statement “To keep blowing even though you do not feel anything is coming out” described a moderate or serious issue. This could be addressed by having an analogue or digital display of flow in ml/s on the screen to give patients feedback on their expiratory rate during the manoeuvre.

After several forced expiratory manoeuvres, fatigue can begin to take a toll on patients, and additional manoeuvres are of little added value^5^. Thus, the FVC manoeuvre is a challenge for some patients, and cooperation and effort become indispensable elements for an acceptable result. Hence, to overcome these hurdles, the PFT induction jacket was invented as a small step towards making the process effortless and productive for both patients and assisting medical practitioners.

The device is enabled with the capability to elicit the initial “diving reflex” or physiological gasp for inspiration^8,9^ and is also capable of providing circumscribing chest compression for expiration. These are brought about by cold water and circumscribing air pressure, respectively; the jacket worn by the participant is supplied with an adequate amount of cold stimulus and circumscribing air pressure to bring forth a physiological gasp response for inspiration and assistive expiration during the equipment. Proper canalization of this response has helped reduce difficulties in obtaining acceptable FVC manoeuvres.

Standards that are developed and updated from time to time should not limit the quest for continual improvement in the quality of lung function measurements and innovation in applying new technology^10^. This jacket was designed to prove the concept that spirometry manoeuvres can be automated, and successful results can be obtained in fewer attempts. However, the criteria for performing clinical spirometry were not followed strictly. According to the American Thoracic Society (ATS/ERS), the spirometry standards are the minimum criteria that must be met for clinical spirometry, which may not be sufficient for all settings, such as research or occupational surveillance^11^. Standards and consensus recommendations are presented for manufacturers, clinicians, operators, and researchers with the aims of increasing the accuracy, precision, and quality of spirometric measurements and improving the patient experience^5^. Continuing research on innovative analyses that may improve diagnoses or lead to earlier diagnosis in at-risk persons is important, and new methods of measuring volume and flow are strongly encouraged^5^. Using the jacket in the suggested application and upgrading its design, we have tried to answer as many questions as possible (the traditional IMRaD format is followed in this article, however we have used a frequently asked question pattern in the discussion section).

## Methods

**Figure 1:**
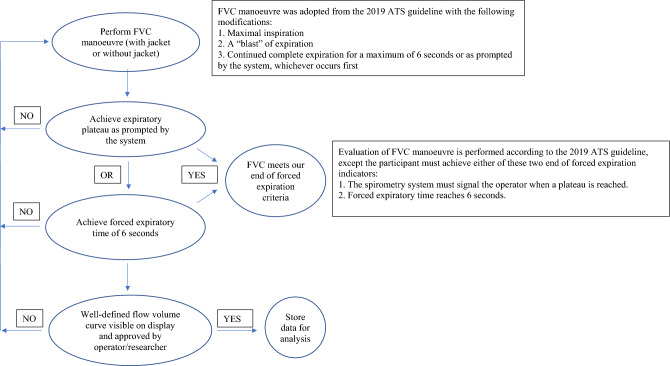
Flow chart outlining the spirometry adopted in this study based on the 2019 ATS/ERS statement.

Spirometry was performed according to the 2019 ATS guidelines for indications, relative contraindications, laboratory details, hygiene and infection control, equipment, patient details and patient preparation but did not follow the FVC manoeuvre completely. To keep both groups comparable, the FVC manoeuvre was restricted to three phases: (1) maximal inspiration, (2) a “blast” of expiration, and (3) continued expiration until the spirometry system signalled that a plateau was reached or forced expiratory time (FET) reached 6 s, whichever occurred earlier (Fig. 1).

### Equipment

- Computerized spirometer (RMS Helios 401)
- PFT induction jacket
- Emergency kit (atropine, adrenaline, ibuprofen, hydrocortisone with required syringe and nebulizer with Asthalin and Budecort respules)

### Design and use of the PFT induction jacket

**Figure 2:**
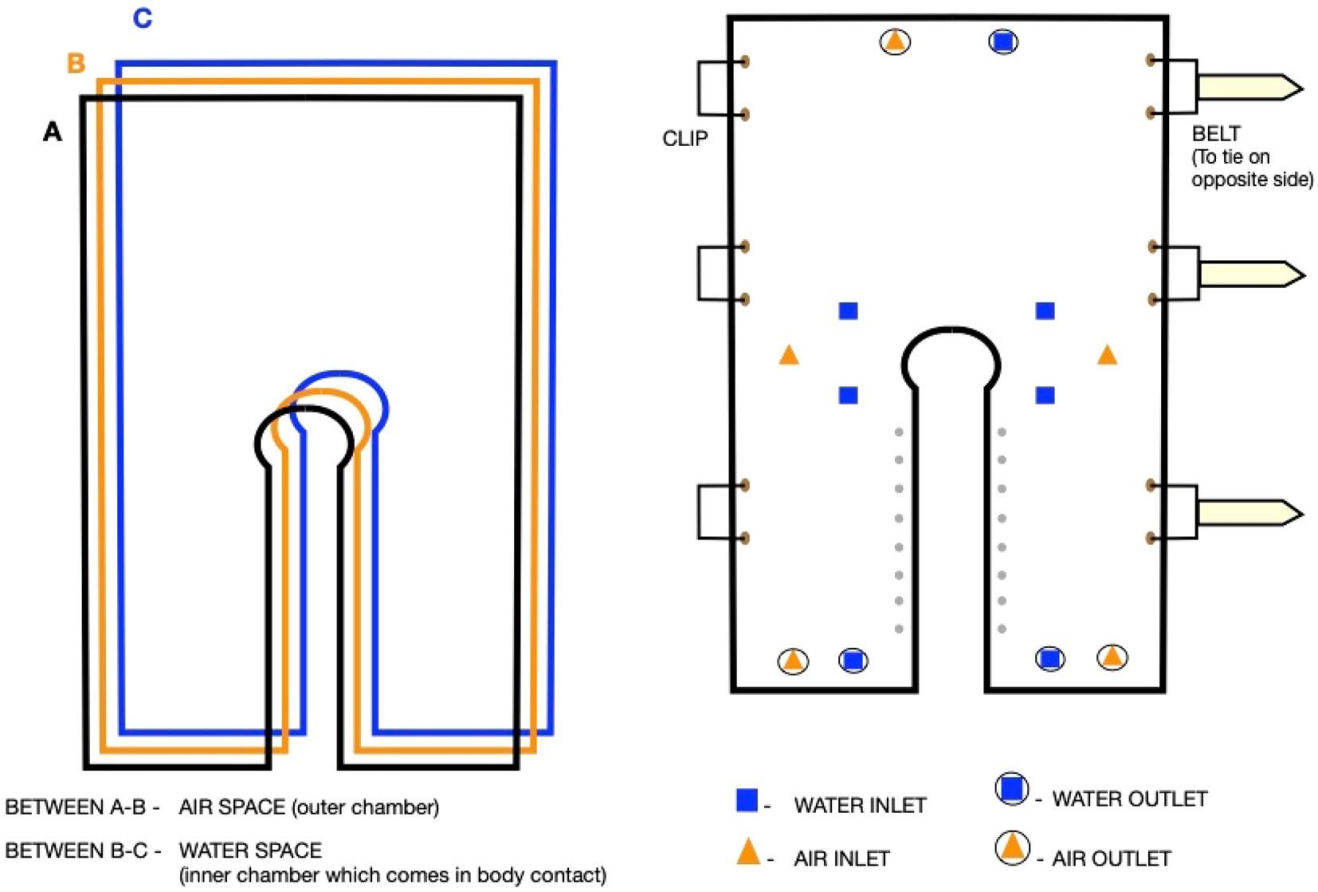
Design of the PFT induction jacket.

**Figure 3:**
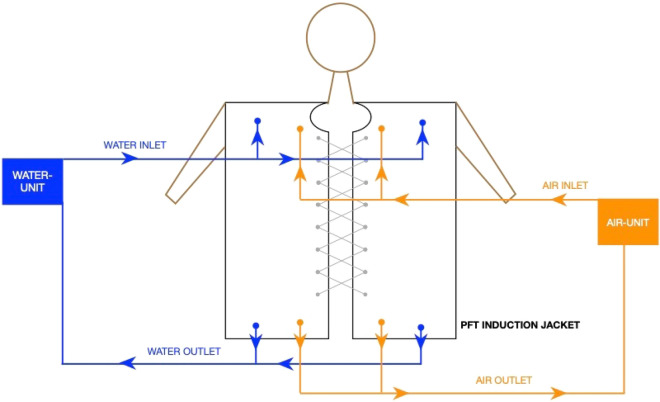
Functionality of the PFT induction jacket.

The Fig. 2 explains the basic framework of the jacket, which is made up of three layers of PVC sheet sealed together to form a single jacket. It is worn in such a way that the water circulates in the inner chamber (which comes in contact with the body), whereas air circulates in the outer chamber. It has belts and clips that are adjustable as per the requirements. There are valves on the jacket through which the water and air enter separately with the help of connecting pipes between the water unit and air unit (refer to Fig. 3 for the schematic including the water unit and air unit). To prevent ballooning of the jacket and to allow the water to travel predominantly on the upper torso, the inner chamber was created with zig-zag folds (not shown in Fig. 2).

### Use of the PFT induction jacket

The Fig. 3 summarizes the basic structure and the outline of the procedure. The participant wore a contact jacket that mostly covered the chest and abdomen. Through the jacket, the desired cold stimulus was provided using cold water at 10 °C in the inner layer of the jacket, which came in direct contact with the skin, to elicit a physiological gasp response by stimulating small receptors that are present on the skin all over the body; however, their maximum concentration is on the upper torso.

The water unit had chilling and pumping components. When the device was switched on, the water was cooled to the desired temperature in a chilling device, while the pumping component forced the cold water into the jacket through an attached pipe. The water was triggered with the initiation of inspiration, which also aided in a proper inspiratory gasp. Similarly, air was pumped under pressure through the air unit, which was connected to the jacket’s outer layer with a pipe, when the participant started expiration after the gasp. Hence, pressurizing the chest aided in expiration. The water and air entered from the top of the jacket and left the jacket from the bottom. The water jet was pushed for approximately 3 s, and pressurized air was pushed for 6 s to maintain the inspiratory:expiratory time ratio.

After obtaining physicians’ approval to participate in the research, thirty willing first-year medical undergraduate male participants who were free of any cardiopulmonary illness (arrhythmia, MI, dyspnoea, pedal oedema, asthma, cold, sinusitis, allergies, bronchitis, and otitis), on no medications, and did not smoke or undertake vigorous exercise one hour before testing were enrolled in the study after providing written informed consent. The protocols were submitted to and approved by the Institutional Ethics Committee for Human Research (IECHR) Medical College and SSG Hospital (Ethics Committee Re-Registration No. ECR/85/Inst/GJ/2013/RR-19). All methods were performed in accordance with the principles of the Declaration of Helsinki. The data collection was performed using a pretested semistructured study instrument.

The study was conducted at the research lab, Department of Physiology, Medical College Baroda. After ensuring proper rest and privacy, the participants performed the spirometry test (using the RMS Helios 401) (http://www.rmsindia.com). To overcome learning bias, the enrolled participants were randomly divided into 2 equal groups. The first group performed spirometry without the aid of the jacket during the first visit and then with the jacket during the second visit on the next day, whereas the second group performed spirometry with the aid of the jacket during the first visit and without the jacket during the second visit on the next day.

### Instructions for performing spirometry without a jacket

The operator (a researcher in this study) demonstrated the appropriate technique to perform spirometry prior to the actual performance by the participant. After ensuring hand sanitation and a zero-flow level, maintaining proper posture, and applying the nose clip, the participant was instructed to insert the mouthpiece such that their lips were sealed around the mouthpiece to prevent any air leakage and instructed to breathe normally or easily. As demonstrated, the participant inspired completely and rapidly with a pause of ≤ 2 s at total lung capacity and expired with maximal effort until the spirometry system signalled the operator/researcher when a plateau was reached or the forced expiratory time (FET) reached 6 s, whichever occurred earlier.

### Instructions for performing spirometry with a jacket

Three researchers were required to conduct spirometry using a jacket (since this was a concept jacket that was not yet automated). Successful performance of spirometry using the jacket required coordination between the three researchers. The first researcher controlled the computer, signalled the other two researchers and assisted the participant in performing the manoeuvre. The second researcher controlled the water unit, and the third controlled the air unit. The first researcher explained the functioning of the jacket prior to actual performance. Researchers helped the participant don the jacket over a bare chest (please view the multimedia file for more details). After ensuring the participant’s comfort and other spirometry steps as explained in the instructions for performing spirometry described above, the participant was instructed to insert the mouthpiece and to breathe normally. The researcher simultaneously observed the patient and the computer display during the test. Just prior to the end of tidal expiration, the first researcher signalled the second researcher to inject cold water into the jacket, which led to the inspiratory gasp, as evident by the graph display and eyebrows raising, eyes widening or head quivering of the participant. At the peak of inspiration when the participant started to expire, the first researcher signalled the third researcher to supply air into the jacket, which provided circumscribing pressure to assist expiration. Expiration progressed until the spirometry system signalled the researcher when a plateau was reached or the forced expiratory time reached 6 s, whichever occurred earlier. The jacket was sanitized after every use.

To evaluate the manoeuvre, most of the criteria described in the 2019 ATS/ERS spirometry technical statement were adopted, except we accepted only one criterion for recognizing a satisfactory end of forced expiration: either the system provided an indicator when the end of forced expiration was reached or the forced expiratory time reached 6 s and maximal inspiration after the end of forced expiration had not been performed (Fig. 1). We opted for a six-second cut-off based on large adult population studies that found that more than 95% of subjects who expired for longer than 6 s achieved a plateau^12,13^. The maximal inspiration after the end of forced expiration step was omitted as the jacket and the body would be of the same temperature (~ 15 degrees) due to the initial inspiratory jolt, thus failing to elicit the required gasp, and although the performance of a maximal forced inspiration is strongly recommended, its absence does not preclude a manoeuvre from being judged acceptable unless extrathoracic obstruction is specifically being investigated^5^. Moreover, the jacket is not equipped for fast removal of water, which would result in additional weight of the jacket due to the second shock.

Repeatability criteria were also not adopted in this study. Rather, the first attempt that the operator/researcher determined to be appropriately well-performed based on the flow volume curve on the system display was considered best for successfully performing spirometry and was analysed. However, the maximum number of manoeuvres that a participant could perform was kept the same as that recommended by the 2019 ATS/ERS statement, i.e., eight attempts. The manoeuvre was not repeated as per the criteria of the 2019 ATS/ERS statement since the application of the concept was to simplify the criteria of repeating the manoeuvre 3 times. Rather, we suggest that with the use of jackets, the best results can be obtained with a minimum number of manoeuvres without dependency on patient cooperation. If there are no prior observed FVC values in the current testing set, then the FVC provisionally meets the end of forced expiration acceptability criteria^5^.

According to the 2019 ATS/ERS statement, after following the repeatability criteria, the data were graded (A to F, starting from the best performance) to indicate the level of confidence that the spirometry results represent the best that the patient was able to do at the time of the test and the probability that an equivalent value would be achieved if the test were to be repeated. Some patients may not be able to meet the criteria for acceptability and repeatability that are necessary for a grade of A, but nevertheless, their results may be clinically useful when given a grade of U (i.e., “usable”). Since the repeatability criteria were not adopted in this study, technically, the data collected in this study are of grade E or U.

Conventionally, the largest FVC and the largest FEV1 observed from all of the acceptable values are reported (or largest usable values if none are acceptable), even though they may not necessarily come from the same manoeuvre. In this study, we have reported the FVC and FEV1 (and other parameters) from the same manoeuvre that has been accepted. Adopting the above changes means that performing the manoeuvre with and without a jacket can be compared.

### Ethical approval and informed concent

This study was performed in line with the principles of the Declaration of Helsinki. Approval was granted by the Institutional Ethics Committee for Human Research (IECHR) Medical College and SSG Hospital, Baroda, of the Maharaja Sayajirao University of Baroda, Vadodara, Gujarat. Written informed consent for publication was obtained.

## Results

Conventionally, the operators must visually inspect the performance of each manoeuvre for quality assurance before proceeding with another manoeuvre. The results depend on the encouragement and coaching of the operator. The operator must have the ability to override the acceptability designation. Thus, the final results used for interpretation are based on operator feedback, which confirms the acceptability and repeatability of FVC manoeuvres^5^.

This study has partially adopted the 2019 ATS/ERS statements, and the advocacy of results would be erroneous at this stage. However, for a pilot project to automate the FVC manoeuvre, the results can be considered to prove the concept. The details of the study participants are given in Table 1.

**Table 1:** General description of participants.

Variable	Mean	Range	SD
Age (years)	19.73	19–21	0.64
Height (m)	1.71	1.62–1.87	0.06
Weight (kg)	60.47	48–87	8.39
BMI (kg/m^2^)	20.60	17.01–26.67	2.25

### Comparison of spirometry results with and without a jacket

All spirometry parameters were recorded for comparison of participant performance with and without a jacket. All the data obtained were entered in MS Excel. As paired quantitative data were used, a paired t test was used to check whether the PFT results by both methods (with and without a jacket) differed significantly. This comparison is summarized in Table 2.

**Table 2:** Comparison of spirometry results with and without the jacket.

Parameter	Without jacket (mean)	%Predicted without jacket	With jacket (mean)	% predicted with jacket	*t* value	*P* value
FVC (L)	3.83	101.33	3.96	104.47	0.850	0.3986
FEV_1_ (L/s)	3.32	100.63	3.37	102.37	0.429	0.6698
FEF_25–75_ (L/s)	3.97	78.63	3.81	78.27	− 0.621	0.5367
PEFR (L/s)	6.78	70.37	7.05	73.13	0.949	0.3467
PIFR (L/s)	4.67	–	5.02	–	0.777	0.4400
FIF_50_ (L/s)	4.55	–	4.79	–	0.543	0.5891

Comparing the spirometry results with and without the jacket, the difference was not statistically significant. Hence, there was no difference between the spirometry results without the jacket and with the jacket.

The above data were also plotted on a Bland‒Altman plot to analyse the agreement between the two methods. The plot shows a scatter diagram of the differences against the mean of the two measurements. The limits of agreement are defined as the mean difference ± 1.96 SD of the differences. These limits do not exceed the maximum allowed difference between the methods; hence, the two methods appear to be in agreement. Refer to the supplementary information 1 for this analysis.

### Comparison of the number of trials required to complete spirometry with and without a jacket

**Figure 4:**
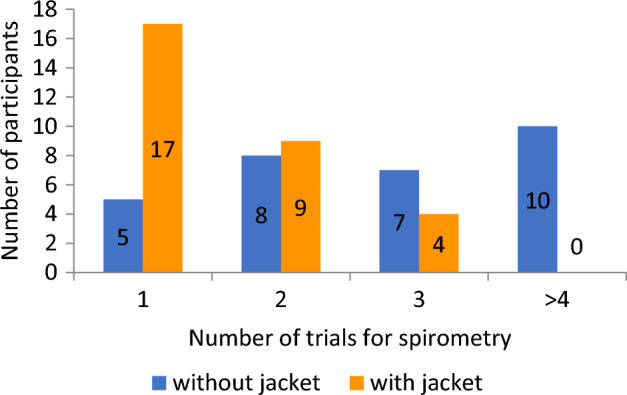
Comparing the frequency of spirometry trials with and without the jacket.

As shown in Fig. 4, the median ± IQR (Q_3_ − Q_1_) number of trials that a participant had to undergo to obtain an acceptable FVC manoeuvre was 3 ± 2 (4 − 2) without a jacket and 1 ± 1 (2 − 1) with a jacket, and this difference was statistically significant (Wilcoxon signed rank test, z = 4.129, *p* < 0.0001), which shows the usefulness of the jacket in performing spirometry and decreasing the effort related to the number of attempts.

## Discussion

The results show that using the jacket as assistance for inspiration and expiration automates the effort and reduces the number of trials required in conducting the FVC manoeuvre. The change in the values of the variables was insignificant, but once a similar study is conducted with a greater number of participants, the results will be more conclusive. Hence, specific investigations of spirometry requirements will result in the proper and specific use of this jacket. Furthermore, implementation of the following discussed suggestions (refer to Supplementary Information 2- Designs and Calculations) may replace or aid body-box plethysmography with jacket plethysmography.

Considering the background of this study, many questions emerge that we have tried to answer.

Q1. In clinical practice, we use a spirometer to observe the patient's ability to move air, assessing both neuromuscular function and lung function. What would be the point of adding artificial mechanical assistance to perform spirometry?

Answer: As explained in the introduction, the FVC manoeuvre is not easy for every patient to perform; even in patients with the ability, it is often not performed properly or with full effort. Hence, proper coordination of this response (gasp and assisted expiration using the jacket) will help automate the efforts required by a patient and will also decrease the number of attempts required to obtain a desired and acceptable manoeuvre.

Additionally, the advanced design suggested in the supplementary information 2 (please refer to the file ‘Design and Calculations’) proposes that the forced expiration would be proportional to the inspiratory efforts. Thus, by mimicking the physiology of forceful expiration, the doubt of confounders in interpreting PFT results can be eliminated, with the additional benefit of measuring residual volume.

Q2. Why are all the subjects male, and why is the sample size small?

Answer: The jacket requires multiple people to operate, and the fact that a bare chest is required limits its use on female participants. Additionally, as explained above, this is a pilot project with the aim of automating spirometry manoeuvres. Hence, to prove this concept with a minimalistic approach, we proceeded with thirty healthy males only.

However, definitive investigative studies regarding the jacket’s suggested advancements and its use in different age groups, types of diseases and sexes are needed.

Q3. Why is cold water at a temperature of 10 °C used for the procedure, and what is the ideal temperature for gasp response?

Answer: The water temperature that comes in direct contact with the body should be 10 °C because at this temperature, the gasp response is maximal. This gasp is equivalent to vital capacity (at 26 °C, the gasp response is approximately 2 L, while at 10 °C, it is approximately 3 L)^9,14^.

Q4. What are the dimensions and distributions of the cold receptors?

Answer: Cold receptors are small spots on the nerve endings that are densely distributed in the upper torso region^15^.

Q5. What are alternative sources for cold stimulation?

Answer: The following alternative sources can be used for cold stimulation:

Peltier thermode – An electric device used to generate cold stimulation. This alternative is an expensive method, and it is very slow to reach the desired temperature.

Cool air—The rate of heat transfer from the body using cool air is 25% less than that of water.

Liquid nitrogen—This alternative is effective but not advisable since accidental leakage can cause severe health hazards.

Q6. What should be the duration of exposure for the inspiratory phase and expiratory phase?

Answer: Inspiratory cold shock and expiratory pressurization are maintained manually in the PFT induction jacket, i.e., cold shock is administered with the start of inspiration for 2 s, while chest compression is administered with expiration for 6 s.

For the expiratory phase, according to the 2019 ATS/ERS spirometry statement, the spirometry system must signal the operator when a plateau has been reached or the forced expiratory time (FET) reaches 15 s. However, to prevent glottic closure and avoid syncope, the effort partway through the manoeuvre (after 4 s)^16,17^ has been reduced even though it gives higher expiratory volume and is not considered a true maximal forced expiration.

The results can be improved if the above timing of induced inspiration and assistive expiration is controlled and executed by computer software in synchronization with the participant’s ventilation^1^.

Q7. How much external pressure is required for assisted expiration?

Answer: According to the tank respirator reference, there is a need for only + 5 cm of H_2_O to obtain assisted expiration^18^. However, in our jacket, we used 70–80 psi (4921.49–5624.56 cm of H_2_O) for effective circumscribing pressure in the jacket. The reason for such a high pressure requirement is that the jacket has a very thin and malleable outer layer, which leads to ballooning; hence, most of the pressure is dissipated on the outer side of the jacket. Additionally, the chest volume decreases with the advancement of expiration. Thus, even though the air unit generates a constant pressure, the jacket exerts a stronger force during the beginning of expiration, which dissipates as chest volume decreases and ballooning of the jacket occurs.

In an advanced jacket, a pressure release valve can be fitted into the jacket for additional safety. We also suggest the addition of pressure sensors in the jacket, which can sense the pressure in the jacket before the start of inspiration and at the end of the inspiratory gasp. This raised end inspiratory pressure in the jacket should be maintained throughout the assisted expiration (application of Boyle’s law as performed in body-box plethysmography; please refer to the file ‘Design and Calculations’ in the Supplementary Information 2).

Q8. What is the weight of the device? Is there any effect of this weight on the pulmonary function test?

Answer: The weight of the device is 1.81 kg (4 lb) without active flow of air and water and 2.95 kg (6.5 lb) with active flow. The results of the present study showed that there was no effect of weight on young and healthy participants.

Many studies have reported the impairment of respiratory functions by external weight. For example, a study by Collins et al.^19^ showed the effect of body fat distribution on pulmonary function tests. In that study comparing pulmonary function tests between patients with a waist-hip ratio less than 0.950 (lower body fat distribution) and subjects with a waist-hip ratio of 0.950 or greater (upper body fat distribution), FVC, FEV1, and TLC were significantly lower in the patients with upper body fat distribution. Stepwise multiple regression analysis was performed using all anthropometric variables and age, which generated predictive equations that included the biceps skinfold thickness for residual volume (RV) and TLC. This suggests that upper body fat distribution may be associated with a modest impairment of lung volumes in normal and mildly obese men. Studies by Marabotti et al.^20^ and Schellart and Sterk^21^ reported the cardiovascular and respiratory effects of the neoprene wetsuit in nonimmersed divers. Pertaining to respiratory effects, twenty-four (24) healthy divers were evaluated by spirometry under basal conditions while wearing a full neoprene wetsuit. Regarding pulmonary function, a significant reduction in vital capacity (− 2%; *p* < 0.001) and expiratory reserve volume (− 25%; *p* < 0.001) and a significant increase in inspiratory capacity (9%; *p* < 0.001) and tidal volume (25%; *p* < 0.05) were observed. Their results supported their hypothesis that neoprene elastic recoil, possibly due to compression exerted on the chest, might affect respiratory function.

With reference to the advancement suggested in Question 9, the impairment of respiratory functions due to weight can be minimized.

Q9. What will be the effects of induced assisted respiration on the flow-volume curves of a participant?

Answer: For any given individual during expiration, there is a unique limit to the maximal flow that can be reached at any lung volume. This limit is reached with moderate expiratory efforts, and increasing the force exerted during expiration does not increase the flow^1^.

This can be understood by a simple example where we suppose that the lung is contained in a thorax with a volume that can be changed by a piston. The piston is formed by the respiratory muscles, which help create negative pressure and positive pressure for inspiration and expiration, respectively. Because the lungs are elastic, they drive the flow and play a vital role in holding the compliant bronchi open even during critical narrowing during forced expiration. In our case, the jacket plays the part of the piston. Provided that the participant has no neuromuscular disorder, this induced assisted respiration via the jacket will mimic the physiological pattern and lead to better results, as expiratory muscle strength is boosted by assisted expiration by the jacket. However, in this study, the results showed that there was no significant change in FEV1 or FVC when using the jacket.

Additionally, regarding the pressure management suggested in Question 7, we also suggest that the jacket can be divided into three main parts, each of which can be further divided into anterior and posterior segments (please refer to the file ‘Design and Calculations’ in the Supplementary Information 2). One part can cover the abdomen, and the other two can cover the lower and upper chest. The purpose is to provide successive compression starting from the abdomen and moving through the lower thorax and the upper thorax, which may minimize the issue of critical narrowing. However, additional investigations are required to prove this hypothesis. The segments can be suspended using a sliding stand, which can help split the weight of the jacket.

The benefit of the three-part design would be in the use of the jacket; the subject can sit in the middle, and the segments on the stand can be moved to be in contact with the body, i.e., the body can be sandwiched between the segments. By doing so, in addition to reducing the duration of the procedure, the cumbersome way to put on and take off the jacket, as shown in the video clip (Supplementary video 1), can also be eliminated.

Q10. What will be the repeatability of the response?

Answer: Humans are highly adaptable. With repeated exposure, the body becomes habituated to cold stress; for example, exposure to cold water for as short a time as three minutes in a 10-min shower will attenuate the cold-shock response by as much as 20–30%^22,23^.

Q11. What are the emergency problems associated with the use of cold shock and external pressure?

Answer: Respiratory responses to cold water immersion have been covered well by Datta and Tipton^9^. This study reported that immersion of an unprotected body in cold water produces a large and rapid decrease in skin temperature, which, in turn, evokes the initial responses to cold immersion, given the generic name “cold shock”. The response comprises inspiratory gasp, hypertension, and hyperventilation. Additionally, neuronal mechanisms suggest that, apart from anticipatory anxiety, cold shock may result in cardiac arrhythmias and cardiac arrest in susceptible individuals, such as those with long QT syndrome.

This setup is a simulation of only the initial part of the cold shock. That is, the participant, breathing freely (without holding their breath), is exposed to cold water for only a few seconds without being immersed, which is associated with the initial physiological effects of cold shock. However, there should be an emergency kit comprising antihypertensives, antiarrhythmic drugs, and defibrillators present in proximity. Since there is only momentary exposure to cutaneous cold stimulation, the abovementioned complications are much less likely to develop. This is also supported by the poor evidence of complications at such a low dose of exposure^9^.

The complications of external pressure administered by mechanical chest compression devices during cardiopulmonary resuscitation include skin or skeletal injury and even life-threatening complications, such as mediastinal bleeding and injuries of the heart, aorta, lung, liver, spleen and stomach^24^.

However, in our jacket, the compression elicited can be compared with the compressions generated by high-frequency chest wall compression (HFCC) devices^25^. These devices are vests that are connected by two tubes to an air-pulse generator, which inflate the vest with a constant positive pressure with a 15–20 Hz frequency of air pressure oscillations. These devices are used as portable mechanical methods of self-administered chest physiotherapy, often required for assisting mainly cystic fibrosis patients with expectorating dislodged bronchial secretions while actively huffing and coughing intermittently during use^26–28^. Our jacket provides chest compression with the intention to assist with a single expiration.

Nevertheless, a question arises regarding the safety of the jacket’s use in a patient with emphysema who has air trapping.

Only definitive investigation of its use among such patients can determine the amount and pattern of permissible pressure required to assist expiration. The jacket can be a useful tool for inducing gasps, e.g., half of the manoeuvre can be automated; for the second half of the manoeuvre, the jacket should be used with caution.

In our present study, no complications or catastrophic events were observed.

Q12. How much time was required to perform spirometry using the jacket?

Answer: The actual performance of the FVC manoeuvre using the jacket requires the same amount of time as conventionally performing it. However, donning the jacket is time consuming. Thus, spirometry took ~ 20–25 min for each participant to perform, which is clearly more time consuming than the conventional method. However, by implementing the suggestion in Question 9, the operational difficulties in the use of the jacket can be minimized.

Q13. What kind of material should be used for the jacket?

Answer: For the inner and middle layers, the material should be durable, malleable and a good conductor of heat. The material must be washable or sterilizable.

For the outer layer, a tough and lightweight material can be used (such as the material used in suitcases).

Q14. How can the jacket be sealed at various places, such as the arms, abdomen, and neck?

Answer: Any flexible and airtight collar can be used for this purpose, as attempted by Bruggink et al.^29^ for leak-free head-out plethysmography in mice. Since an advanced technique is required to achieve this feat, the present device does not have this setup.

Q15. How can the results be improved?

Answer: Computer software, which can better sync the delivery of cold shock and assisted expiration with the patient’s breathing pattern, can increase the accuracy (also mentioned in Question 6). Using software can remove the need for manual delivery, which requires perfect coordination among multiple staff to achieve.

Application of the other suggested advancements can also aid in improving the results. In this article, we are more focused on advocating the concept rather than the results.

Q16. What are the advantages and disadvantages of the jacket?

Answer: After discussions with the patients, doctors, and technicians, we decided to make modifications based on the advantages and disadvantages mentioned in Table 3.

**Table 3:** Advantages and disadvantages with the jacket and without the jacket.

	Spirometry without jacket (conventional way)	Spirometry with jacket
Advantages	No gender bias	Saves time by aiding the manoeuvre Effort for training of the patients reduced Reduced number of attempts to perform the acceptable manoeuvre will allow for investigation of more patients in less time Reduces patient effort-related errors in the report Aiding stimuli are purely physiological
Disadvantages	Time-consuming due to many initial weak efforts, especially by noncompliant patients Too many attempts may cause fatigue and reduced efforts during the manoeuvre Vigorous coaching is required	Donning the jacket is time consuming Needs further research in different age groups, genders, and diseases before using it for diagnosis Emergency kit should be available to deal with cold stimulus-related responses, if any

Q17. What are the limitations of the study?

Answer: Apart from the small sample size and gender-specific use mentioned in Q2, the study did not strictly follow the latest spirometry guidelines. Keeping the disadvantages (Table 3) in mind, the jacket has not been tested on certain patients (patients with lung function impairment, asthenic patients, elderly patients, low BMI patients, etc.). Thus, the results must be interpreted with caution. Furthermore, many questions remain unanswered, such as whether the jacket influences air trapping and the effect of its application in plethysmography with residual volume (RV) and RV/total lung capacity (TLC) measurements. The results in patients may be completely different. The jacket in its present form cannot be used in clinical practice; however, the suggested advancement is that the jacket can be a useful aid for less compliant patients (especially non cooperative patients such as paediatric, mentally challenged, inpatients who cannot perform the normal spirometry, claustrophobics), and can be a substitute for body box plethysmography.

Q18. Is there a possibility for improvements in the jacket?

Answer: Since the patent was granted (Granted By—The Patent Office, Government of India. Patent No-394704, Application No- 1596/Mum/2014, Date of Filing- 08/05/2014, Date of grant 12/04/2022, Patentee- Dr Prashant Rajdeep), commercialization gates have been opened, which expands the scope. With an advanced design and materials (as suggested in Questions 7, 9, 13, 14, and 15 and in the Supplementary Information 2; Design and Calculations), the jacket is potentially able to convert full-body plethysmography to jacket plethysmography. This provides additional benefits of better compliance and cost effectiveness for the patients.

Therefore, we can conclude that this invention is based on a unique concept that can help reduce the number of voluntary efforts of patients undergoing spirometry. It will not only assist the patients but will also reduce the trial, time, and energy of the medical practitioner in teaching the manoeuvre to the patients. Additionally, with further advancements in the jacket, it has the capability to convert body-box plethysmography to jacket plethysmography.

## Supplementary Information

Supplementary Information 1.

Supplementary Information 2.

Supplementary Video 1.

Supplementary Information 3.

Supplementary Information 4.

## Data Availability

All the data generated or analysed during this study are included in this published article [and its supplementary information files 3 and 4].
