# Supplementary material for: A pilot study for development of a pulmonary function test induction jacket to automate effort in performing the forced vital capacity manoeuvre
Source: Sci Rep. 2023 May 17;13:8004. doi: 10.1038/s41598-023-34930-1 (PMC10189700; doi:10.1038/s41598-023-34930-1)
Supplement: Supplementary file 1 — Supplementary Information 1. [file 41598_2023_34930_MOESM1_ESM.docx]

Plot-1 Bland-Altman plot of agreement between FVC with jacket and without the use of jacket

Difference between FVC with Jacket & without Jacket

Mean of FVC with Jacket & FVC without Jacket

Plot-2 Bland-Altman plot of agreement between FEV_1_ with jacket and without the use of jacket

Difference between FEV_1_ with Jacket & without Jacket

Mean of FEV_1_ with Jacket & FEV_1_ without Jacket

Plot-3 Bland-Altman plot of agreement between FEF_25-75_ with jacket and without the use of jacket

Difference between FEF_25-75_ with Jacket & without Jacket

Mean of FEF_25-75_ with Jacket & FEF_25-75_ without Jacket

Plot-4 Bland-Altman plot of agreement between PEFR with jacket and without the use of jacket

Difference between PEFR with Jacket & without Jacket

Mean of PEFR with Jacket & PEFR without Jacket

Plot-5 Bland-Altman plot of agreement between PIFR with jacket and without the use of jacket

Difference between PIFR with Jacket & without Jacket

Mean of PIFR with Jacket & PIFR without Jacket

Plot-6 Bland-Altman plot of agreement between FIF_50_ with jacket and without the use of jacket

Difference between FIF_50_ with Jacket & without Jacket

Mean of FIF_50_ with Jacket & FIF_50_ without Jacket

The plot shows a scatter diagram of the differences against the mean of the two measurements. The limits of agreement are defined as the mean difference ±1.96 SD of differences. As we can see here, these limits do not exceed the maximum allowed difference between the methods. The two methods are appeared to be in agreement.
