# Supplementary material for: A pilot study for development of a pulmonary function test induction jacket to automate effort in performing the forced vital capacity manoeuvre
Source: Sci Rep. 2023 May 17;13:8004. doi: 10.1038/s41598-023-34930-1 (PMC10189700; doi:10.1038/s41598-023-34930-1)

1. INNER LAYER

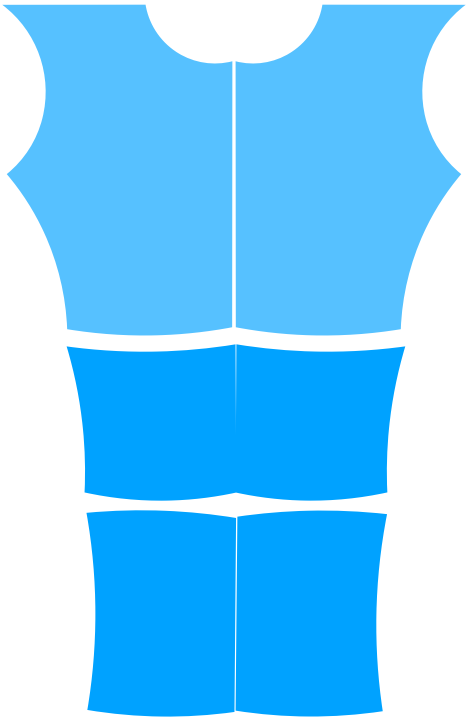

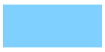

ELASTIC AND
TOUGH SUIT
MATERIAL

2. ARRANGEMENTS OVER INNER LAYER FRONT VIEW

1 UPPER CHEST PIECE

2 LOWER CHEST PIECE

3 ABDOMINAL PIECE

WATER INLETS

WATER OUTLETS

MALLEABLE LIGHT WEIGHT PIPE

STITCHING/ SEALING POINTS

ELASTIC AND TOUGH SUIT MATERIAL

PIECE CONNECTOR (TOUGH MALLEABLE MATERIAL)

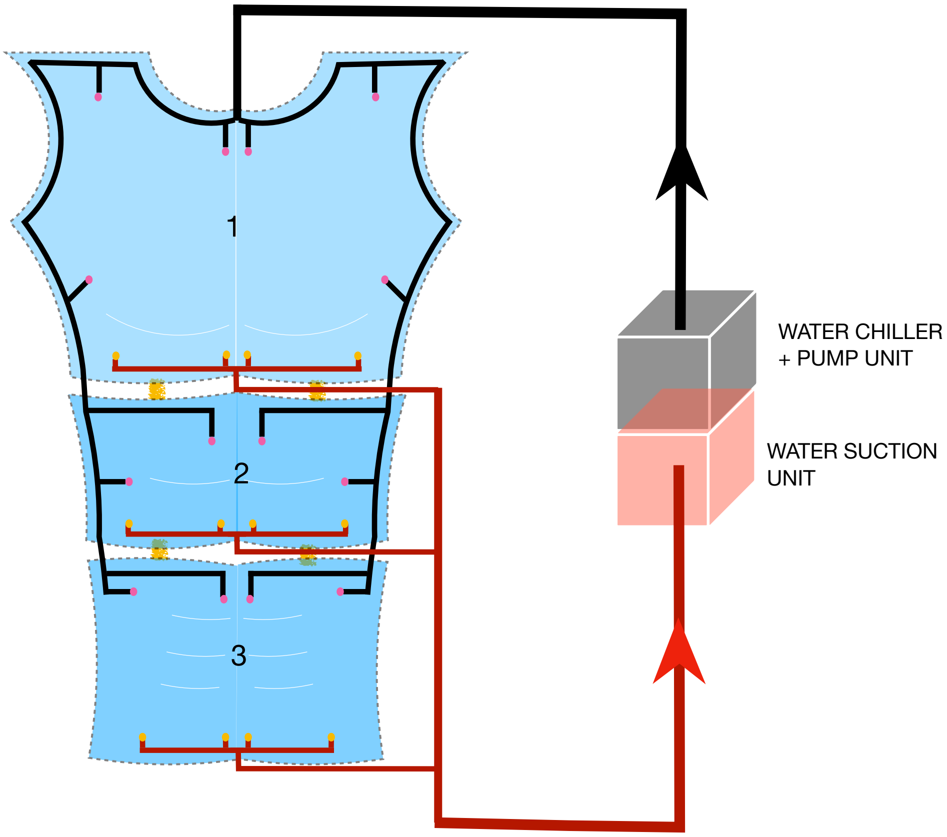

DIRECTION OF WATER FLOW INSIDE SUIT

DIRECTION OF WATER FLOW OUTSIDE SUIT

3. ARRANGEMENTS OVER INNER LAYER BACK VIEW

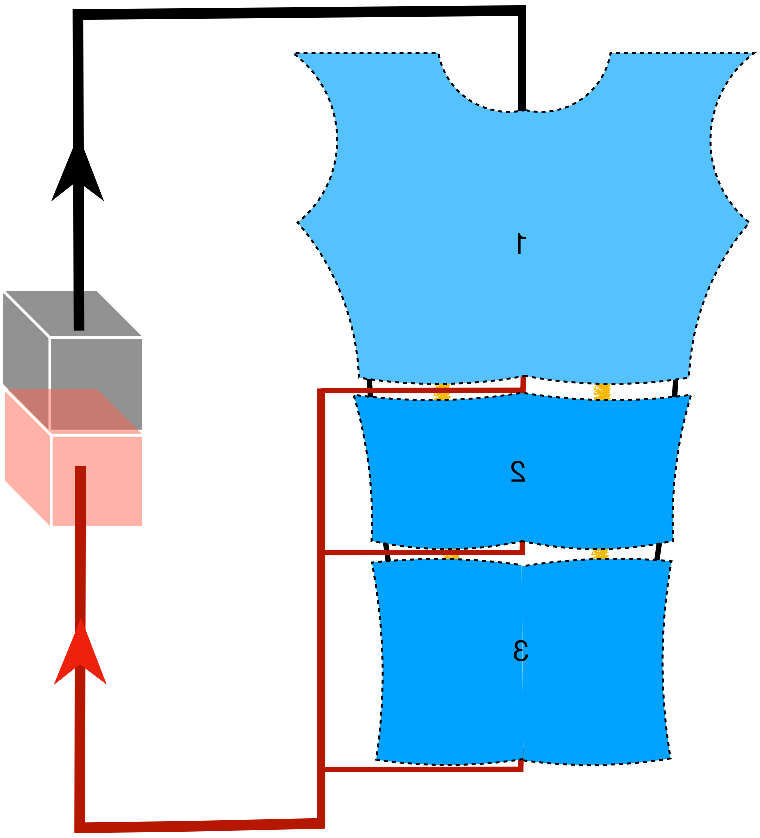

4. ARRANGEMENTS OVER INNER LAYER FRONT VIEW -ANTERIOR AND POSTERIOR PARTS

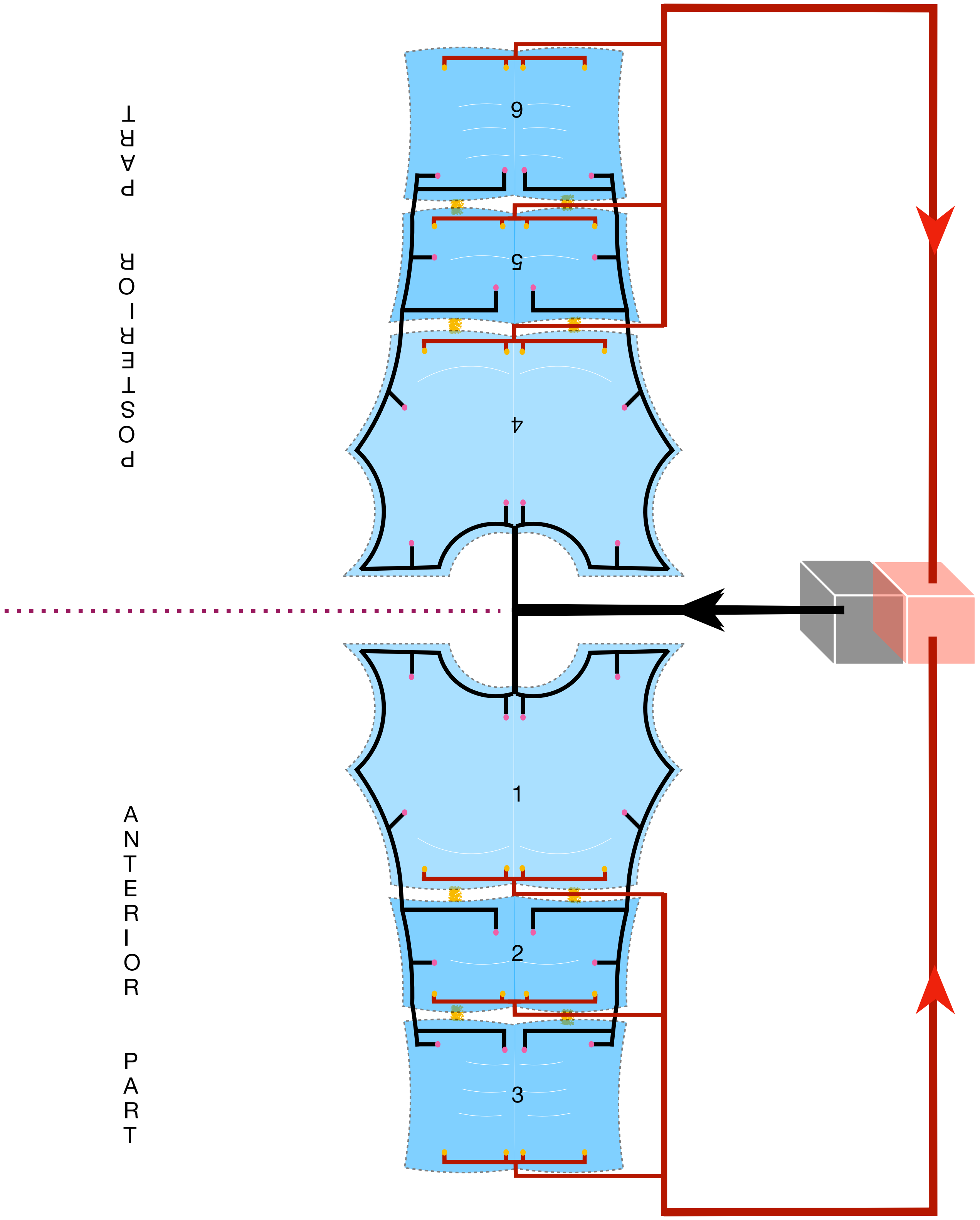

5. ARRANGEMENTS OVER INNER LAYER BACK

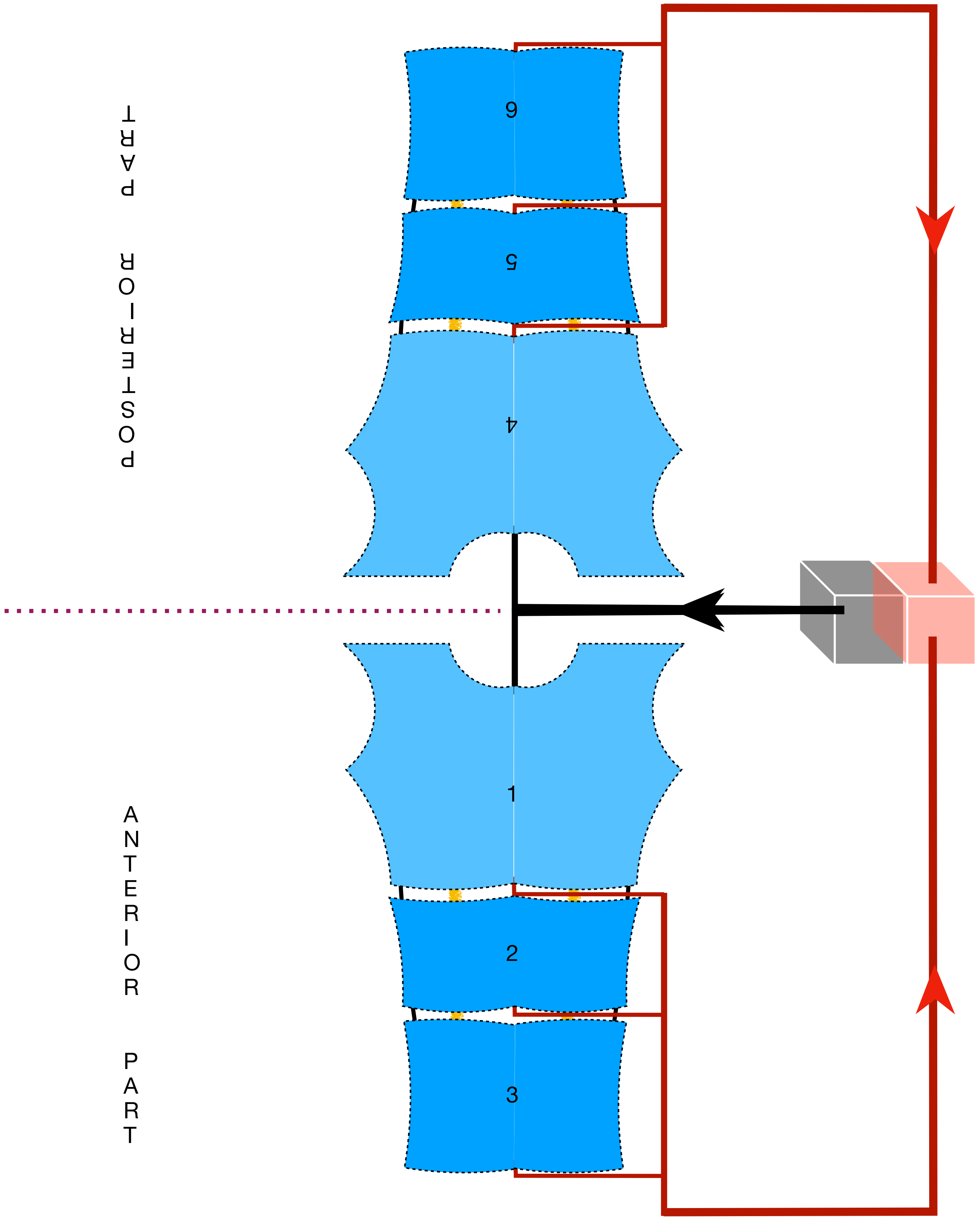

6. MIDDLE LAYER

ELASTIC AND
TOUGH SUIT
MATERIAL

7. MIDDLE LAYER ALL PARTS

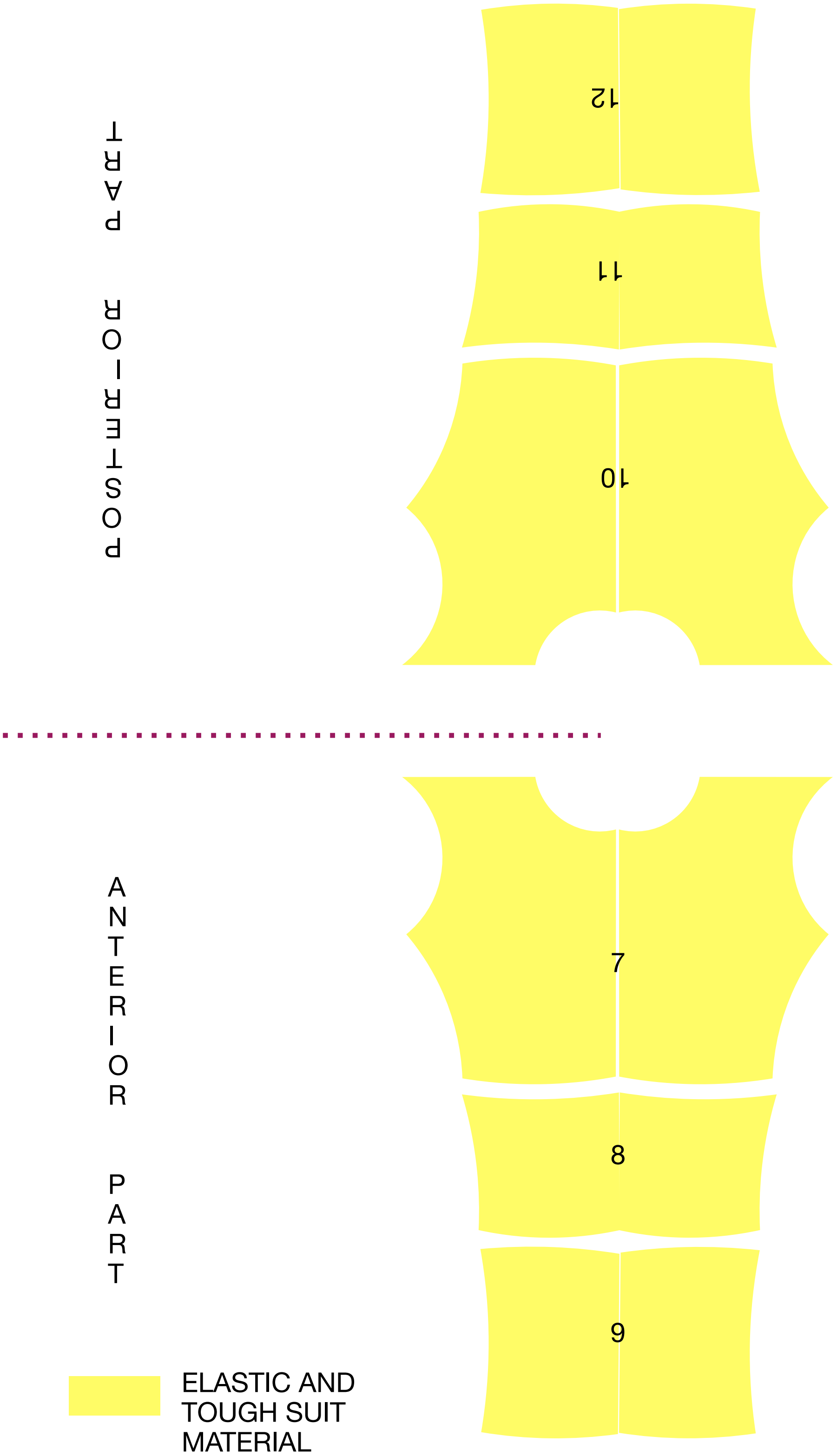

8.OUTER LAYER

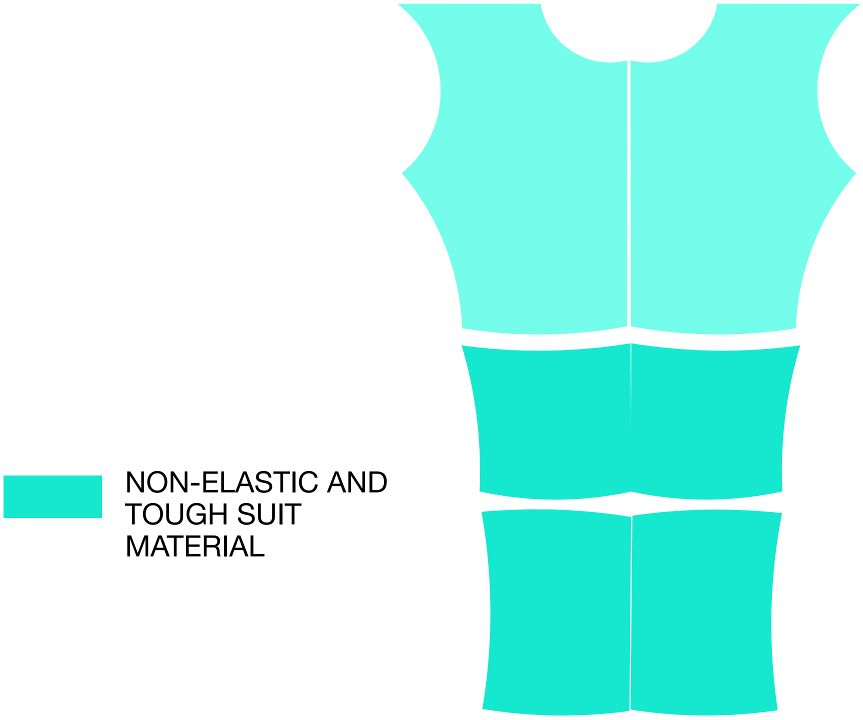

9. ARRANGEMENTS UNDER THE OUTER LAYER BACK SIDE VIEW

1 UPPER CHEST PIECE

2 LOWER CHEST PIECE

3 ABDOMINAL PIECE

AIR INLET ONEWAY VALVE

AIR OUTLET ONEWAY VALVE

MALLEABLE LIGHT WEIGHT PIPE

STITCHING/ SEALING POINTS

NON-ELASTIC AND TOUGH SUIT MATERIAL

PIECE CONNECTOR (TOUGH MALLEABLE MATERIAL)

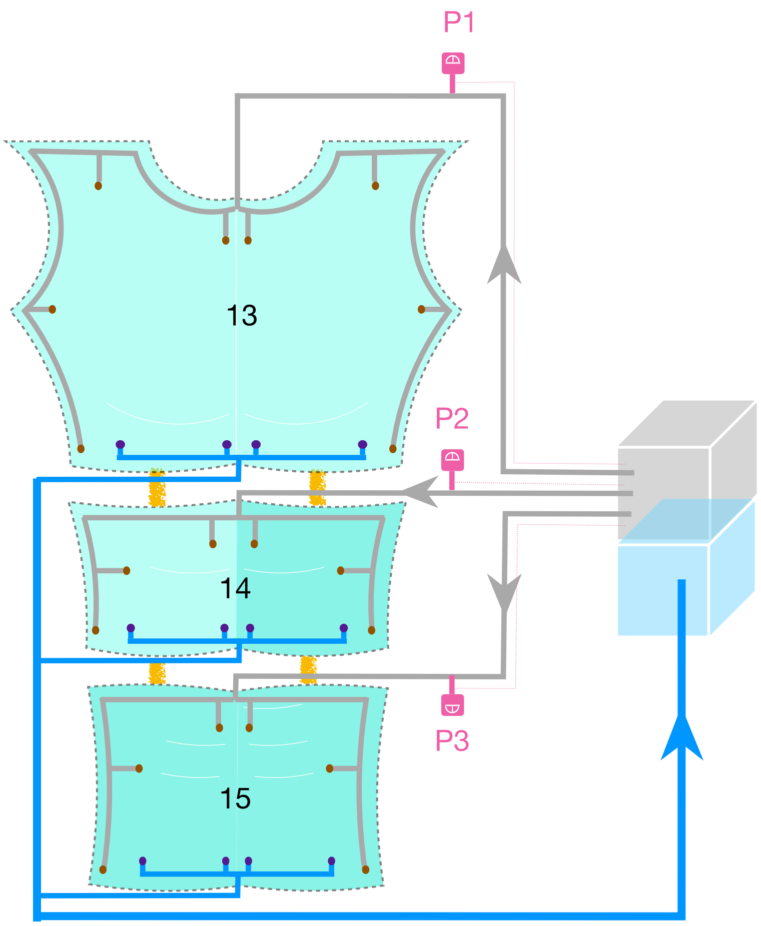

DIRECTION OF AIR FLOW INSIDE SUIT

DIRECTION OF AIR FLOW OUTSIDE SUIT

P

PRESSURE SENSOR
1- UPPER CHEST PIECE BETWEEN PIECE 13 AND 7
2- LOWER CHEST PIECE BETWEEN PIECE 14 AND 8
3- ABDOMINAL PIECE BETWEEN PIECE 15 AND 9
4-UPPER CHEST PIECE POSTERIOR PART BETWEEN PIECE 16 AND 10
5-LOWER CHEST PIECE POSTERIOR PART BETWEEN PIECE 17 AND 11
6-ABDOMINAL PIECE POSTERIOR PART BETWEEN PIECE 18 AND 12

10. ARRANGEMENTS UNDER OUTER LAYER BACK VIEW -ANTERIOR AND POSTERIOR PARTS

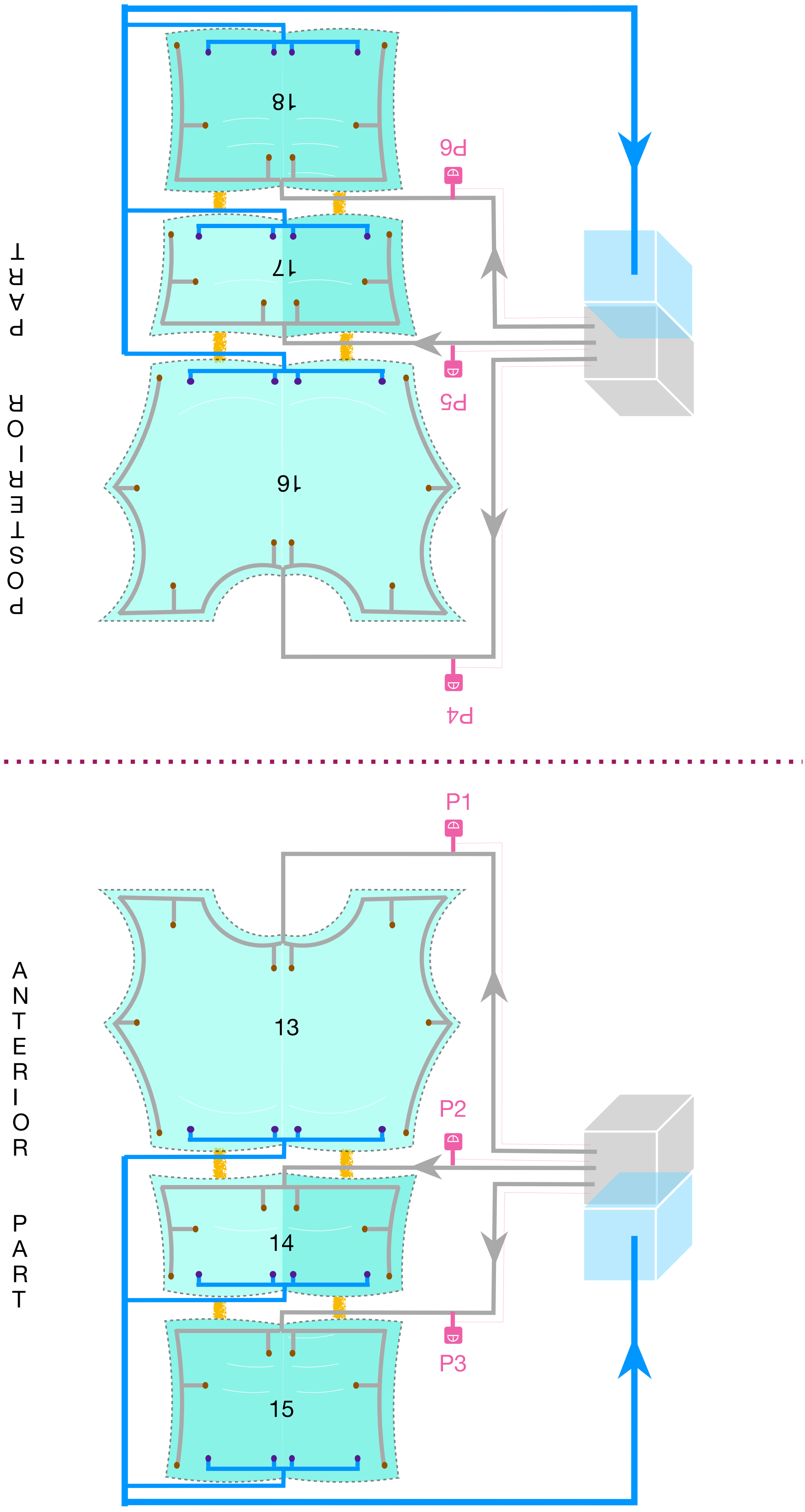

11.STITCHING ALL LAYER BACK VIEW

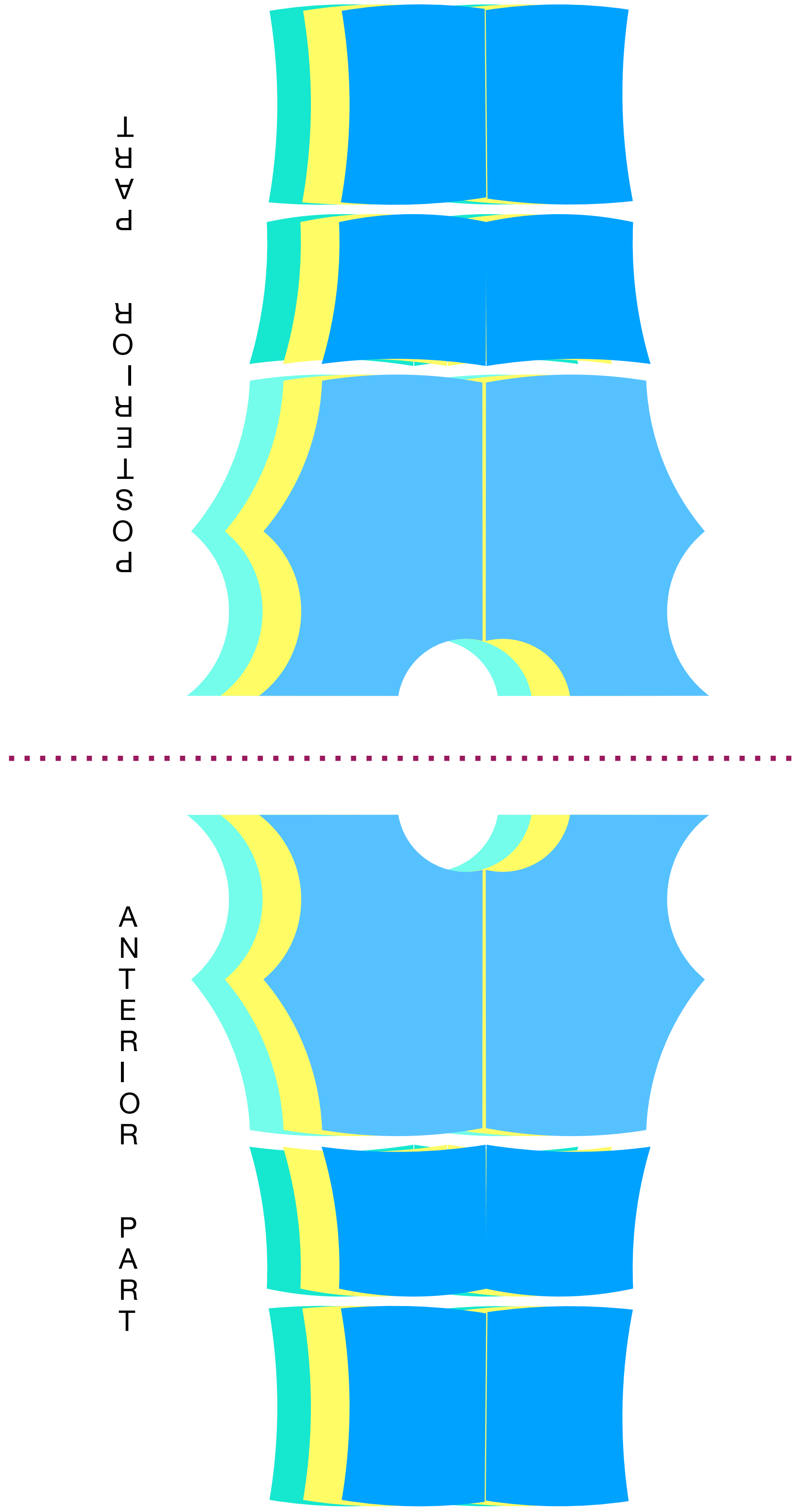

12.STITCHING ALL LAYER FRONT VIEW

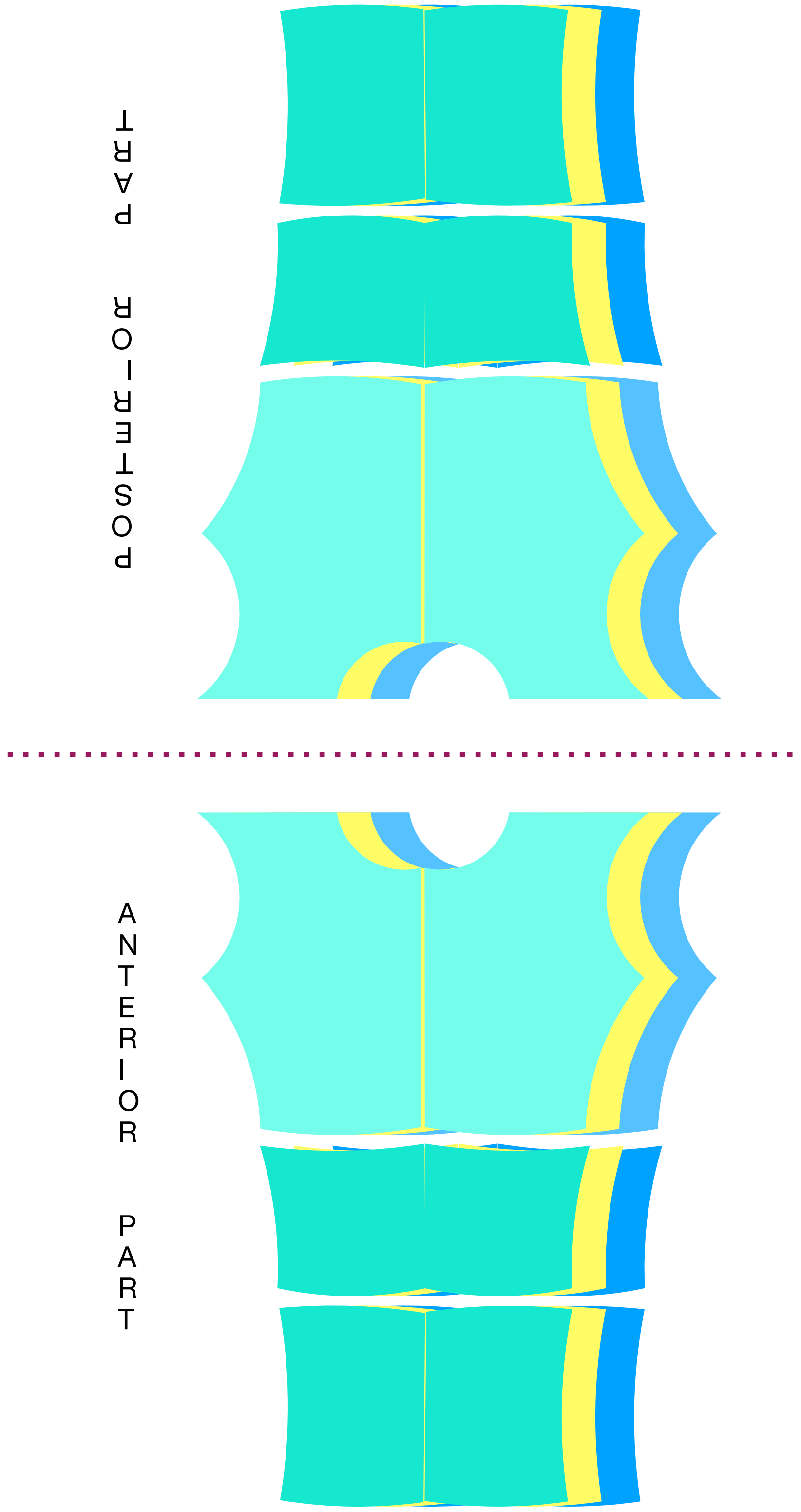

13.TOP VIEW

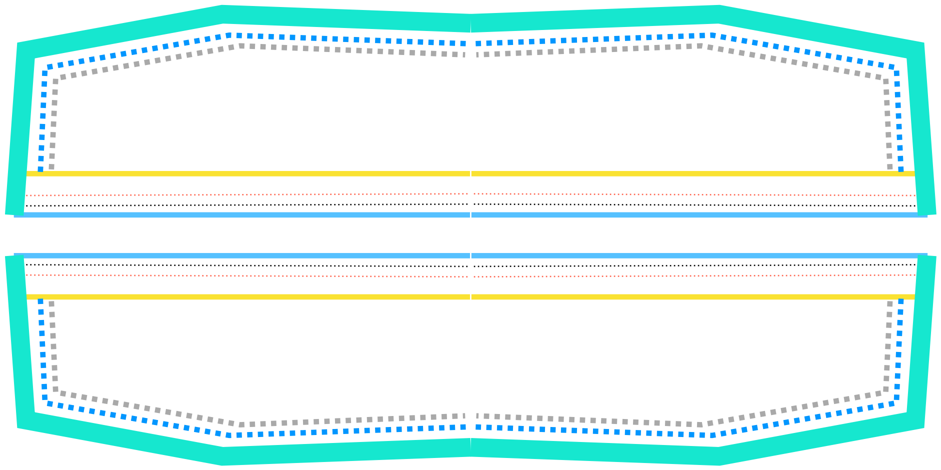

14.TOP VIEW WITH BODY (CROSS SECTION) AND TUBINGS

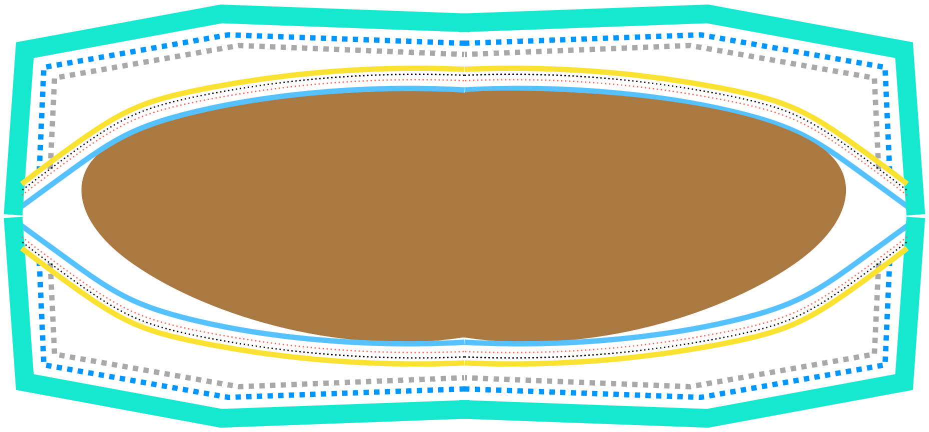

15.TOP VIEW WITH BODY WITHOUT TUBINGS

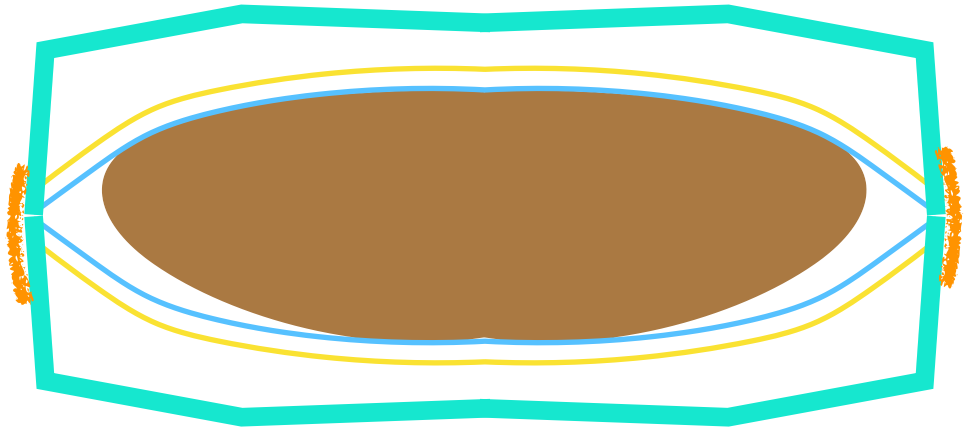

STRAPS FOR TYING ANTERIOR AND POSTERIOR PARTS
(SHOWN IN ONLY ONE PAIR OF ANTERIOR AND POSTERIOR PARTS

16.TOP VIEW WITH SUIT HOLDING STAND WITH BODY WITHOUT TUBINGS

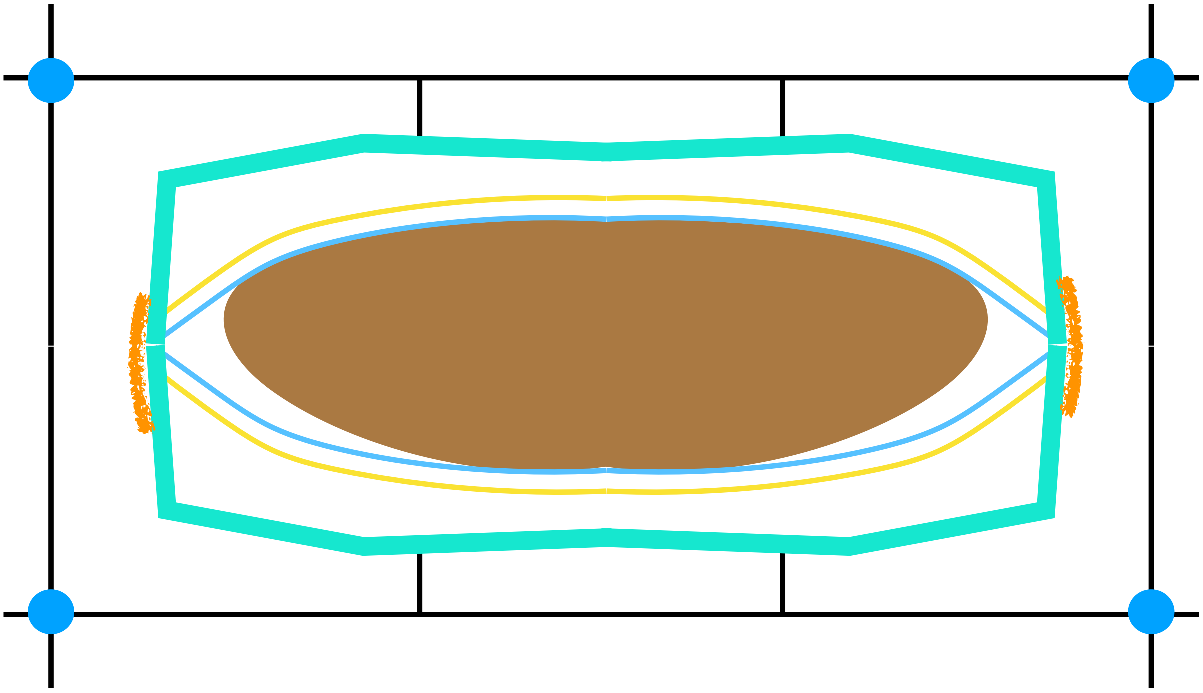

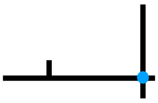

STAND TOP VIEW

17.OBLIGUE VIEW STAND HOLDING SUIT

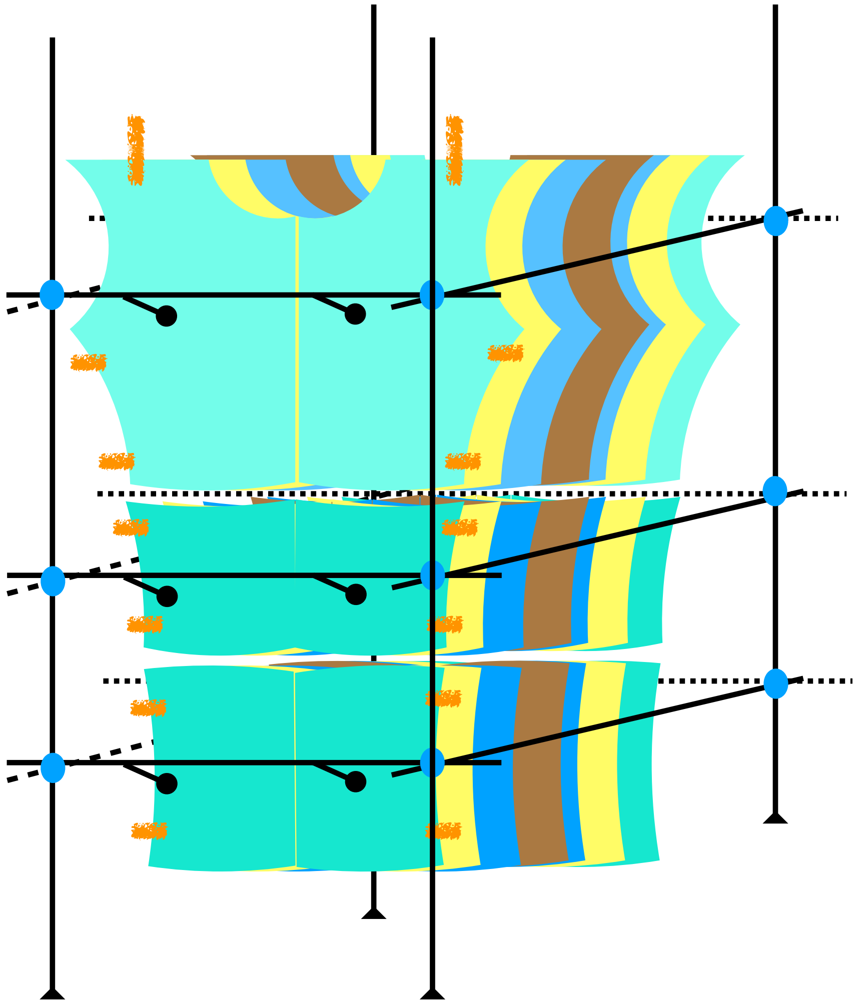

SCREW ALLOWING UP-DOWN AND ANTERIOR POSTERIOR MOVEMENTS

BODY

18. WORKING ALGORITHM AND CALCULATIONS

I- Neutral position after donning jacket.

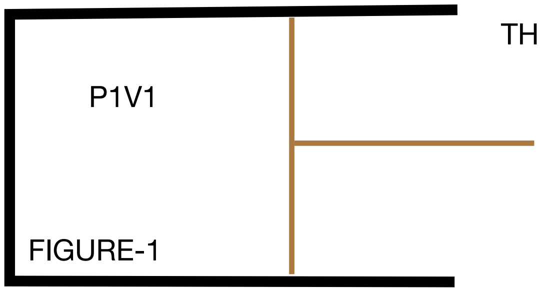

THE BROWN PISTON represents the middle and inner layer which are adhere to the chest wall.

THE BALCK BOX represents the outer layer forming a chamber.

FIGURE 1 shows the position of the chest wall and outer layer AFTER TIDAL EXPIRATION. P1 and V1 are pressure and volume of the chamber in that position.

Estimation of V1

Where, P1 can be measured by pressure sensor in the chamber while V1 can be estimated by the following steps-

Method-1

From the predetermined volume of the chamber (such as for the cuboid LxBxH), subtract the volume of air escaped when positioning a patient.

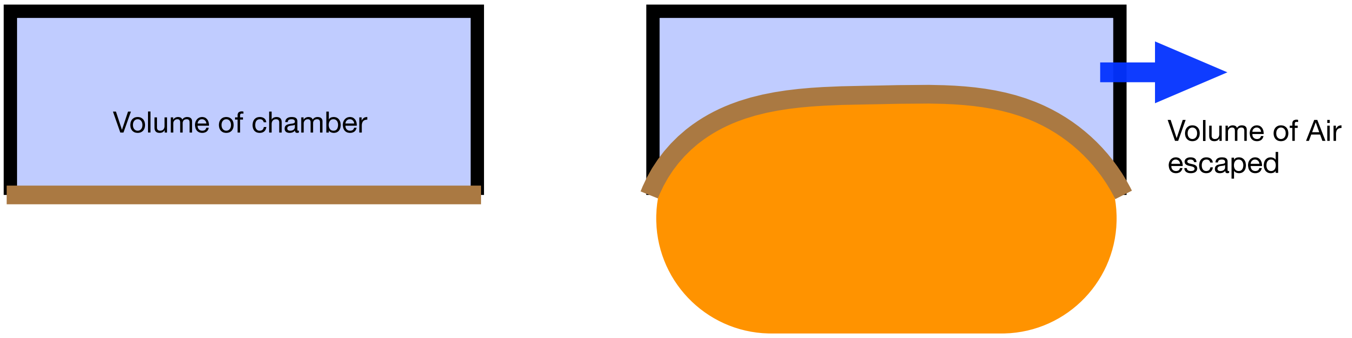

Method-2

All the air in the chamber is sucked out to create a vacuums in the chamber. Then calculating the volume to air required to gradually fill the chamber such that the inner layer just touches the chest wall (as indicated by the patient when the layer touches the chest skin.

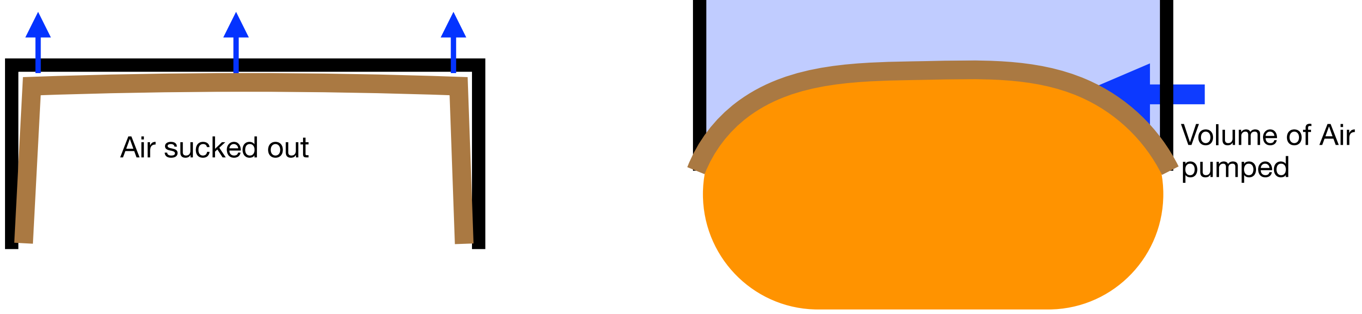

The patient should be instructed to take few tidal respirations so that sensing system can measure the required volume and pressure accurately. (system must consider the maximum volume estimated as V1 during tidal respiration)

II- Position after induced inspiration

At the end of tidal expiration, inspiratory gasp is triggered using cold water in inner chamber. Hence, after vital inspiration the new position will be as shown in figure 2.

Estimation of V2 and P2

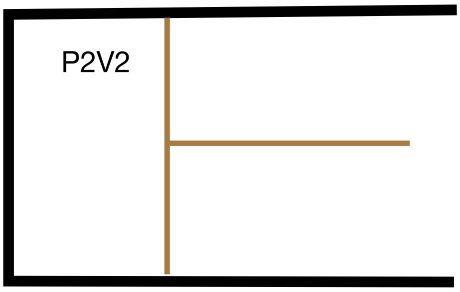

FIGURE-2

Now, as per Boyle's Law

$P1V1=P2V2$

Thus, $V2=P1V1/P2$

Where, P2 can be measured easily by the pressure sensors.

And, $\Delta V_0 = V1-V2$

III- Position after forced expiration

With the end of the inspiratory gasp and beginning of expiration circumscribing air is pressurised to assist expiration. The said amount of pressure is calculated as under.

Estimation of V3 and P3

Thus, after forceful expiration the last position of the manoeuvre will be as shown in figure 3.

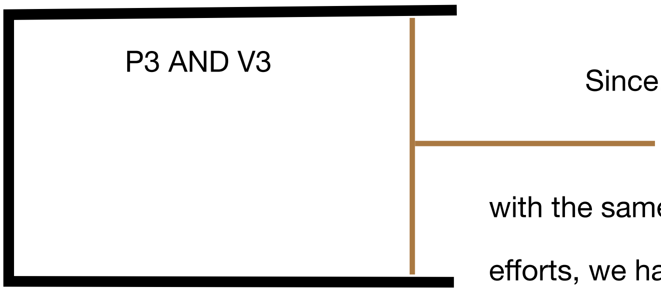

FIGURE-3

Since, we want the final picture to be like figure-3, in which we want to mimic forced expiration with the same pressure as generated during inspiratory efforts, we have to maintain the pressure volume ration. (To mimic forced expiration, in which we want to displace the piston with the same ratio of P2/V2)

i.e., $P2/V2=P3/V3$

Where, $V3= V2 + \Delta V_0 + 40\%$ OF ΔV_0 (or $V1 + 40\%$ OF ΔV_0)

Therefore, P3=P2V3/V2

(40% OF ΔV because required volume displacement over neutral position is nothing but the expiratory reserve volume, which in turn is 40% of inspiratory reserve volume i.e, (ERV/IRV)x100)

Delivery of P3

P3 must be delivered as shown below.

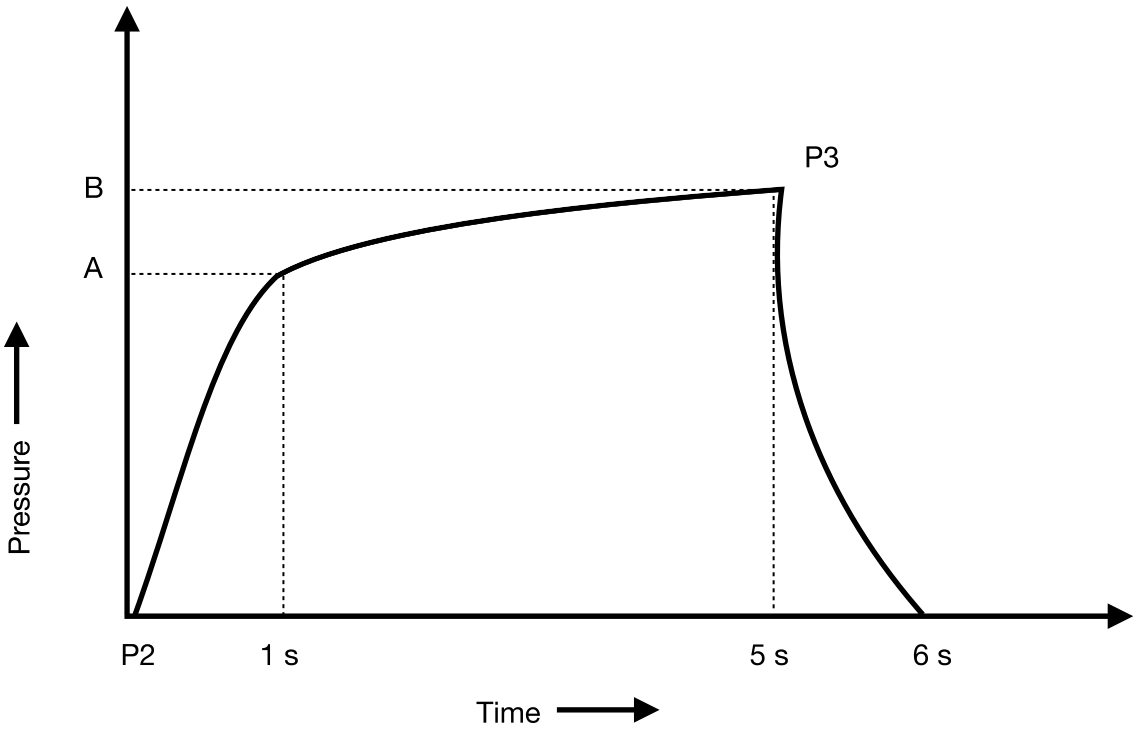

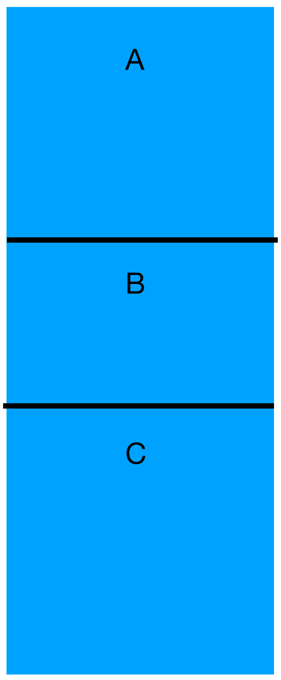

Where, starting from P2 to P3, 80% of the P3 must be delivered in 1st second (A) while rest of the pressure to be delivered gradually over the next 5secs (B), followed by gradual release (till atmospheric pressure/ to open the jacket, not shown beyond P2 in the graph).

Refer figure 4, the calculations for P3 has to be done in all the six chambers, three in front, and three in back. Furthermore, the calculated pressure must be delivered in the respective chamber with marginal gradient starting from the lower segment to upper side. Example, infusion of air to achieve required pressure must start with segment C at time (T) = 0 s and P_c = max (P3). Followed by B after say 300 ms and $P_B = P_c - 2$ (slightly less than P3) and lastly segment A after 600 ms of start in segment C and $P_A = P_B - 2$.

Figure 4

Moreover, the above suggested gradient delivery of pressure in different segments may not be required as the calculated pressure (P3) would be different in each chambers based on the movement of chest wall. However, the sequential compression of, abdominal segment, lower thoracic, and upper thoracic segment may micic physiological forceful expiration. That may also reduces the critical narrowing.

Furthermore, the principles of body box plethysmography can also be clubbed with the jacket so that it can even determine the residual volume. In other words, the box can be reduced to a jacket with additional benefit of assisting patient in performing spirometry manoeuver.

Kindly note - The above proposed designs and calculations have not been checked. Additionally, apart from the suggested shape (cuboid), other shapes (such as, frustum and inverted frustum; as suggested in the jacket design), their sizes and its sequential compression requires an immense research to mimic physiological forceful expiration.

19.ADDITIONAL VIEW LATERAL WITHOUT STAND

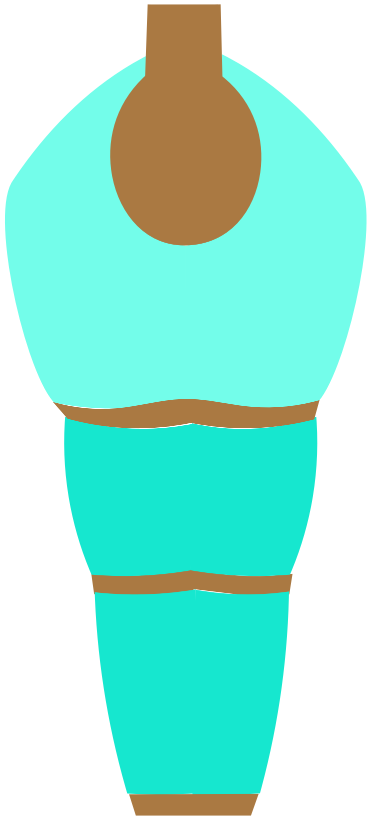

20.ADDITIONAL VIEW SAGITTAL

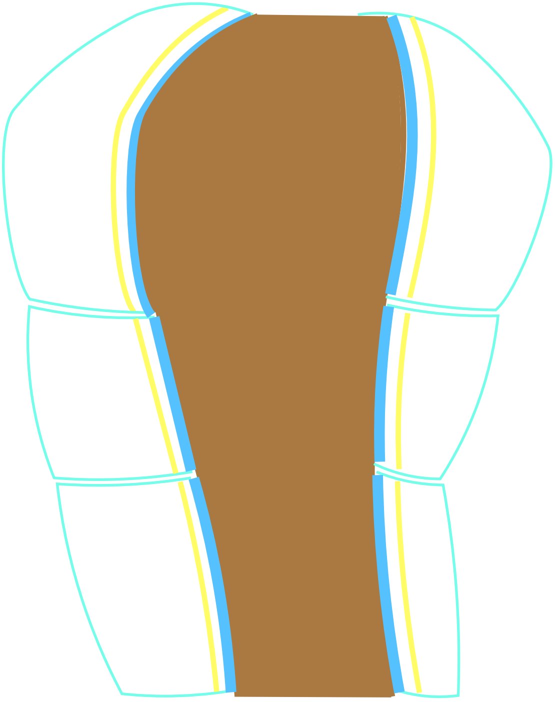

Supplement: Supplementary file 2 — Supplementary Information 2. [file 41598_2023_34930_MOESM2_ESM.pdf]
